# Neuroprotective effects of astaxanthin against oxygen and glucose deprivation damage via the PI3K/Akt/GSK3β/Nrf2 signalling pathway in vitro

**DOI:** 10.1111/jcmm.15531

**Published:** 2020-06-21

**Authors:** Jie Zhang, Changling Ding, Shuping Zhang, Yangyang Xu

**Affiliations:** ^1^ Department of Radiology Binzhou Medical University Hospital Binzhou China; ^2^ Department of pharmacy Binzhou Medical University Hospital Binzhou China; ^3^ Department of Pharmacology Binzhou Medical University Yantai China

**Keywords:** astaxanthin, GSK3β, Nrf2, oxygen and glucose deprivation, PI3K/Akt, SH‐SY5Y

## Abstract

Astaxanthin (ATX), which is the most abundant flavonoid in propolis, has previously shown neuroprotective properties against cerebral ischaemia‐induced apoptosis. However, the mechanisms by which ATX mediates its therapeutic effects are unclear. At present, we explored the underlying mechanisms involved in the protective effects of ATX via the phosphoinositide 3‐kinase (PI3K)/Akt/glycogen synthase kinase 3 beta (GSK3β)/nuclear factor erythroid 2‐related factor 2 (Nrf2) signalling pathway in SH‐SY5Y cells. The PI3K/Akt inhibitor LY294002 and GSK3β inhibitor LiCl were employed in this study. Pre‐treatment with ATX for 24 hours significantly decreased the oxygen and glucose deprivation (OGD)‐induced viability loss, reduced the proportion of apoptosis and regulated OGD‐mediated reactive oxygen species (ROS) production. Furthermore, ATX suppressed OGD‐caused mitochondrial membrane potential and decomposition of caspase‐3 to cleaved caspase‐3, and heightened the B‐cell lymphoma 2 (Bcl‐2)/Bax ratio. PI3K/Akt/GSK3β/Nrf2 signalling pathway activation in SH‐SY5Y cells was verified by Western blot. ATX and LiCl treatment raised the protein levels of p‐Akt, p‐GSK3β, nucleus Nrf2 and haeme oxygenase 1 (HO‐1). However, these protein expression levels decreased by treatment of LY294002. The above in vitro data indicate that ATX can confer neuroprotection against OGD‐induced apoptosis via the PI3K/Akt/GSK3β/Nrf2 signalling pathway.

## 1 INTRODUCTION

**FIGURE 1:**
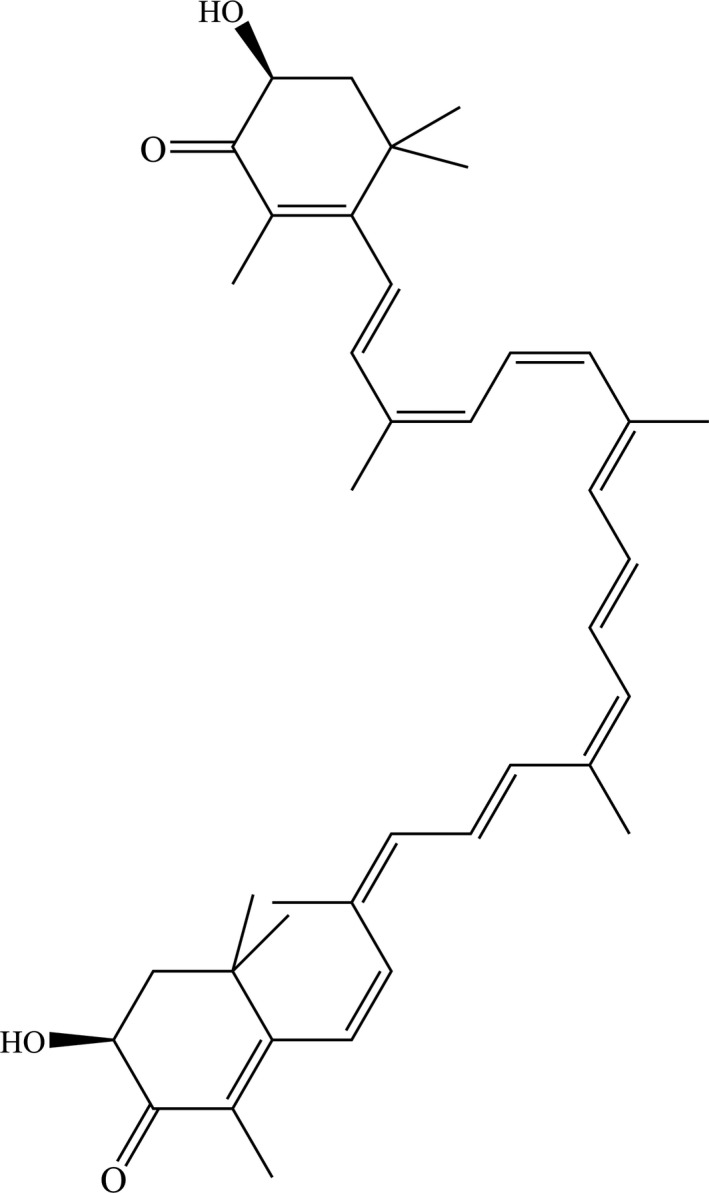
Chemical structure of ATX (3, 3′‐dihydroxy‐β, β′‐carotene‐4, 4′‐dione)

Astaxanthin (3, 3′‐dihydroxy‐β, β′‐carotene‐4, 4′‐dione, ATX) is a xanthophyll carotenoid found in the algae *Haematococcus Pluvialis, Chlorella Zofingiensis* and *Chlorococcum* and the yeast *Phaffia Rhodozyma*. The chemical structure (Figure 1) shows that ATX contains hydroxyl and ketogroups as well as a conjugated polyene chain, which could be lipophilic and hydrophilic, shows strong antioxidative properties and has been reported beneficial for eye, skin and heart health.^1^, ^2^ Previous studies have shown the protective potential of ATX against cerebral ischaemia injury by inducing angiogenesis via Wnt/β‐catenin signalling pathway in vitro.^3^ ATX also shows neuroprotective effects against H_2_O_2_‐induced neurotoxicity with its antioxidant and anti‐inflammatory properties.^4^

Stroke is one of the leading causes of mortality, long‐term disability and morbidity.^5^, ^6^ OGD is a commonly used simulation of ischaemia in vitro with the employment of sodium dithionite and non‐glucose cultural medium.^7^ ROS, which impairs cell organelles with an increased production, might be one of the important factors for neurotoxicity that occurs in cerebral ischaemia.^8^, ^9^ More and more studies show that the benefits of antioxidants in decreasing oxidative stress in neurons and providing protection against cerebral ischaemia. Nuclear Nrf2 is a transcription factor and is considered as a defence regulator against oxidative stress.^10^ Some studies have identified a possible regulating effect between PI3K/Akt, GSK3β, as well as an Nrf2 activation.^11^, ^12^ However, the antioxidant effect of ATX through PI3K/Akt/GSK3β/Nrf2 signalling pathway has not been reported. In the current study, we explored the protective effects of ATX and its molecular mechanism against OGD damage through the PI3K/Akt/GSK3β/Nrf2 signalling pathway at a cellular level.

## 2 MATERIALS AND METHODS

### 2.1 Drugs and reagents

Astaxanthin (3, 3′‐dihydroxy‐β, β′‐carotene‐4, 4′‐dione, C40H52O4: 596.84, Senbeijia Biological Technology, PR China, purity > 98.0%). MTT (3‐(4,5‐dimethylthiazol‐2‐yl)‐2,5‐diphenyltetrazolium bromide) was purchased from Beyotime Biotechnology (Shanghai, China). A PI3‐K inhibitor LY294002 (Calbiochem, USA) and the GSK3β inhibitor LiCl (Beyotime Institute of Biotechnology, PR China) were used in this study.

### 2.2 Cell culture and chemical treatment

The human neuroblastoma SH‐SY5Y cell line was obtained from the Shanghai Saily Biotechologies Co., Ltd. The DNA of the cell lines found to perfectly match the type of cell lines in a cell line retrieval. DSMZ database shows that cells called SH‐SY5Y, corresponding to the cell number 209. No multiple alleles were discovered in this cell line. SH‐SY5Y cells were maintained in Dulbecco's modified Eagle's medium (DMEM, HyClone, USA) supplemented with 10% foetal bovine serum (FBS, HyClone, USA) in an atmosphere of 5% CO_2_ at 37°C.

The OGD model was established with a solution of sodium dithionite (Na_2_S_2_O_4_, Sigma‐Aldrich) at a concentration of 20 mmol/L in glucose‐free DMEM (Gibco), since sodium dithionite could react with oxygen dissolved in the culturing medium.

### 2.3 Measurement of cell viability with methyl thiazolyl tetrazolium (MTT) assay

Cell viability was measured by the MTT (Beyotime Biotechnology, PR China) assay. Briefly, the cells (1 × 10^4^ cells/well) were incubated with null‐vehicle or ATX (5, 10, 20 and 40 μmol/L) for 24 hours and co‐treated with OGD for 3 hours in the continued presence of vehicle or ATX. After incubation, 10 μL of MTT solution (5 mg/mL) was added into each well and the final concentration made up to 0.5 mg/mL. Plates were incubated for an additional 4 hours, and the absorbance at 490 nm was measured in a microplate reader. Per cent viability was defined as the relative absorbance of treated cells compared with untreated control cells.

### 2.4 Determination of Malondialdehyde (MDA) and Superoxide Dismutase (SOD) Production

SH‐SY5Y cells were seeded in 6‐well plates at a density of 4 × 10^5^ cells/well in the culture medium. The cells were incubated with ATX (5, 10 and 20 μmol/L) for 24 hours prior to exposure to OGD for 3 hours. Cells were collected and washed twice with PBS before lysis. After cells being treated with lysis buffer for 30 minutes, the lysates were ultracentrifuged at 12 000*g* for 30 minutes. SOD activity and MDA contents were measured according to the manufacturer's protocol (Kit S0109 and S0131, Beyotime Institute of Biotechnology, PR China).

### 2.5 Determination of ROS Production

ROS production was detected by determining intracellular ROS formation using the DCFH2‐DA (2′,7′‐dichlorofluorescin diacetate) probe. In short, SH‐SY5Y cells were pretreated with ATX (5, 10 and 20 μmol/L) for 24 hours and then stimulated with OGD for 3 hours to induce ROS production. SH‐SY5Y cells were washed twice and then incubated with DCFH2‐DA probe (10 μmol/L) for 30 minutes. Fluorescence staining was visualized using a fluorescence microscope (Olympus, IX71), and fluorescence assays were measured with a fluorescence microplate reader (Tecan) at excitation/emission wavelength of 488/525 nm.

### 2.6 Mitochondrial membrane potential (Δψm) measurement

Mitochondrial membrane potential (ΔΨm) was measured using 2‐(6‐amino‐3‐imino‐3H‐xanthen‐9‐yl) benzoic acid methyl ester (rhodamine 123, Beyotime Biotechnology, PR China), which can diffuse into the mitochondrial matrix and reflect Δψm change. SH‐SY5Y cells (1 × 10^5^ cells/well in 6‐well plates) were incubated with ATX (5, 10 and 20 μmol/L) for 24 hours before incubating with OGD (3 hours) and then incubated with rhodamine 123 (5 mg/mL) for 30 minutes. The SH‐SY5Y cells were collected by centrifugation (1500 *g*, 5 minutes) and resuspended in the proper buffer. The fluorescence intensities of rhodamine 123 were analysed by flow cytometry.

### 2.7 Annexin V‐FITC/PI double‐staining assay

SH‐SY5Y cells with distinct treatments were tested with Annexin V‐FITC/PI double‐staining assay kit. The cells were incubated with various concentrations of ATX (10, 20 μmol/L) for 24 hours and then stimulated with OGD for 3 hours. The cells were carefully collected by trypsin, washed with PBS twice and resuspended in 200 μL of binding buffer at 1 × 10^5^ cells/mL. The processed samples were incubated with 5 μL of in the dark at room temperature for 15 minutes. The samples were detected by flow cytometry (10 000 events were gated in each sample) and evaluated according to the percentage of cells positive.

### 2.8 Measurement of protein expressions by Western blot analysis

SH‐SY5Y cells were seeded in 6‐well plates at a density of 4 × 10^5^ cells/well as described above. To confirm the effects from ATX on cell apoptosis and the PI3K/Akt/GSK3β/Nrf2 signalling pathway, different cell treatments were employed in this study: (a) The SH‐SY5Y cells were pretreated with ATX (5, 10, 20 μmol/L) for 24 hours before incubating with OGD for 3 hours. The levels of bcl‐2, bax, caspase‐3 and cleaved caspase‐3 from the total protein fractions were tested by Western blot. (b) The SH‐SY5Y cells were treated either with 5, 10 and 20 μmol/L ATX for 24 hours or with 20 μmol/L ATX for 6, 12, 24 and 48 hours, and the levels of nuclear and cytosolic Nrf2 were tested by Western blot, respectively. (c) Total protein fractions were extracted from the SH‐SY5Y cells within different ATX (5, 10 and 20 μmol/L) treatments, respectively, and protein expressions of Akt, p‐Akt, GSK3β, p‐GSK3β, Nrf2 and HO‐1 were evaluated. (d) Cells are pretreated with LY294002 (10 μmol/L) or LiCl (10 μmol/L) for 1 hours before incubation with ATX (20 μmol/L, 24 hours) and OGD (3 hours), and protein expressions of Akt, p‐Akt, GSK3β, p‐GSK3β, Nrf2 and HO‐1 were determined.

All groups of cells are carefully collected with scrapers and washed twice with ice‐cold PBS. Total protein extraction and cytoplasmic/nuclear protein extraction were collected using the RIPA and Nuclear and Cytoplasmic Protein Extraction Kit containing protease inhibitor cocktail (Senbeijia Biological Technology). The protein concentration of the supernatant was tested by BCA assay. Proteins at the amount of 40 μg from each sample were separated by SDS‐PAGE and transferred to polyvinylidene difluoride membranes (Millipore, USA). After blocking with 6% non‐fat dry milk in TBST, membranes were incubated overnight at 4°C with primary antibodies against Akt, p‐Akt, GSK3β, Nrf2, HO‐1, Bax, Bcl‐2, cleaved caspase‐3/caspase‐3 (all of which are diluted at 1:2000, from Abcam, UK), antibodies against β‐actin (1:1000, Cell Signaling Technology) and Lamin B1 (1:1000, ImmunoWay, USA), respectively. Membranes were incubated with secondary antibodies for 3 hours at 25°C. Antigen‐antibody complexes were detected, and the bands were analysed by using ImageJ analysis software.

## 3 RESULTS

### 3.1 ATX protects cultured SH‐SY5Y cells against OGD‐induced cytotoxicity

**FIGURE 2:**
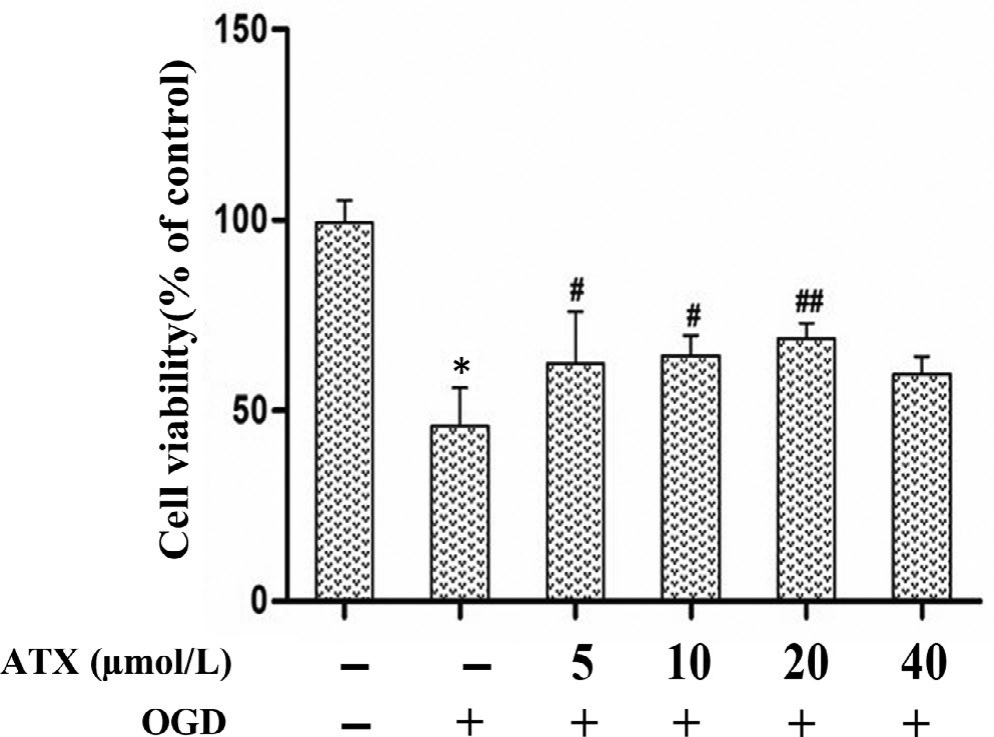
Effects of ATX on cultured SH‐SY5Y cells against OGD‐induced cytotoxicity. MTT assay was performed to detect cell viability after the cells were treated with different concentrations of ATX (5, 10, 20 and 40 μmol/L) for 24 h prior to exposure to OGD for 3 h. Data from experiments were expressed as mean ± SD, n = 5. *P < .01 vs Control group, #*P* < .05, ##*P* < .01 vs OGD group. Significance was determined by one‐way analysis of ANOVA followed by Dunnett's test

The MTT assay was used to detect the effect of ATX on SH‐SY5Y cell viability. As shown in the MTT assay, cell viability markedly reduced after OGD for 3 hours (Figure 2). Interestingly, when cells were incubated with ATX (5, 10, 20 and 40 μmol/L), cell viability significantly enhanced in a concentration‐dependent manner, with peak viability at approximately 20 μmol/L, exposure of ATX (*P* < .05).

### 3.2 ATX decreases intracellular ROS levels and attenuates OGD‐induced oxidative stress

**FIGURE 3:**
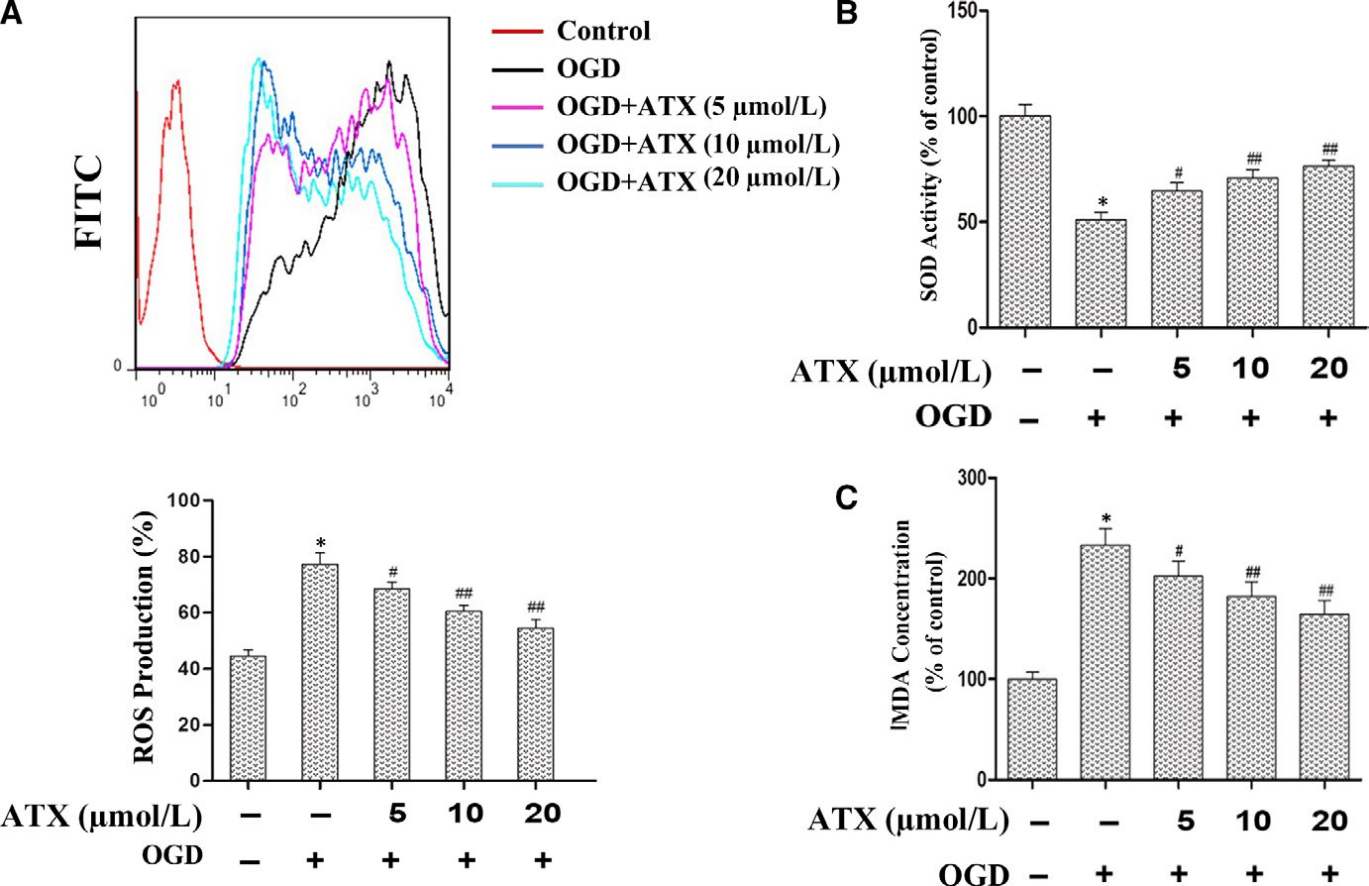
ATX decrease the intracellular ROS level and attenuate oxidative stress induced by OGD. Cells were pretreated with ATX (5, 10 and 20 μmol/L) for 24 h, which was followed by incubation with OGD in SH‐SY5Y cells for 3 h, cells were collected and oxidative stress was assessed by measuring intracellular ROS level, SOD activity and MDA level. A, Intracellular ROS level was determined by flow cytometry with DCFH2‐DA probe, and the results was expressed in counts of FITC(DCF) positive cells in different groups. B, SOD activity and (C) MDA level were expressed as mean ± SD, n = 3. *P < .01 vs Control group, #*P* < .05, ##*P* < .01 vs OGD group. Significance was determined by one‐way analysis of ANOVA followed by Dunnett's test

To determine whether ATX suppresses OGD‐leaded oxidative stress, we detected intracellular ROS levels, SOD activity and MDA levels. As shown in Figure 3, after inducing OGD, intracellular ROS and MDA levels significantly elevated (Figure 3A,C). However, SOD activity significantly reduced compared with the control (Figure 3B), indicating that OGD induced oxidative stress. When the cells were pretreated with ATX (5, 10, 20 μmol/L) for 24 hours and exposed to OGD for 3 hours, MDA levels and intracellular ROS remarkable reduced compared with the OGD group (*P* < .05). Pre‐treatment of ATX (5, 10, and 20 μmol/L) notably increased SOD activity in OGD‐induced SH‐SY5Y cells (*P* < .05).

### 3.3 ATX inhibits OGD‐induced SH‐SY5Y cell apoptosis

**FIGURE 4:**
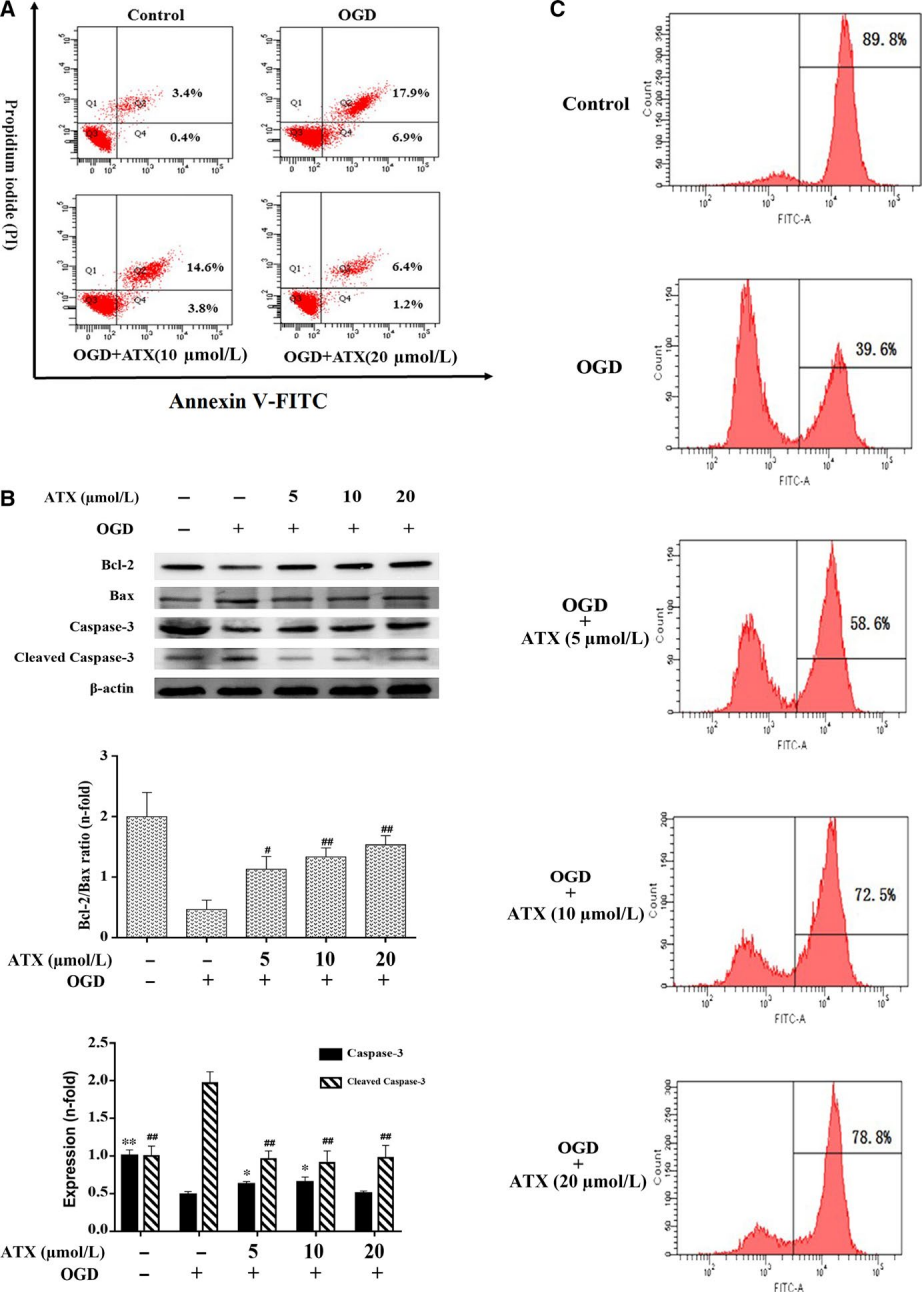
Effect of ATX on the apoptosis of SH‐SY5Y cells. A, Apoptotic induction was determined by Annexin V/PI double‐staining assay after ATX treatment (10 and 20 μmol/L) for 24 h. B, Bcl‐2 Bax, caspase‐3 and cleaved caspase‐3 protein expressions were obtained by Western blot analysis. C, Cells treated with ATX (5, 10 and 20 μmol/L) for 24 h prior to exposure to OGD for 3 h were incubated with rhodamine 123 and evaluated by flow cytometry. The cell percentages in the right section of fluorocytogram indicated the number of mitochondrial membrane potential (Δψm)‐collapsed cells. Results were expressed as mean ± SD (n = 3). * #*P* < .05, ** ##*P* < .01 vs OGD group. Significance was determined by one‐way analysis of ANOVA followed by Dunnett's test

Annexin V‐fluorescein isothiocyanate/propidium iodide double‐staining assay was conducted to determine whether ATX inhibits OGD‐induced cell apoptosis. As shown in Figure 4A, OGD resulted in a substantial increase in apoptosis. After 3 hours of incubation with OGD, 24.8% of cells underwent apoptosis, whereas only 3.8% apoptosis was observed in the control group. However, when cells were pretreated with ATX (10 or 20 μmol/L), apoptosis rates were suppressed to 18.4% and 7.6%, respectively. As shown in Figure 4B, the protein levels of Bax increased and the expression trend of Bcl‐2 protein was opposite by OGD. ATX (5, 10 and 20 μmol/L) pre‐treatment inhibited the up‐regulation of Bax and down‐regulation of Bcl‐2 on treatment with OGD. All these data suggested that ATX treatment partially restrained the decrease in the Bcl‐2/Bax ratio observed after OGD treatment (*P* < .05).

As shown in Figure 4B, the expression of cleaved caspase‐3 increased on OGD, and pre‐treatment with ATX (10, 20 μmol/L) decreased the OGD‐induced enhancement of cleaved caspase‐3 in a concentration‐dependent manner (*P* < .01). Meanwhile, compared with OGD, the expression of caspase‐3 in dose groups (5, 10 μmol/L) was up‐regulated, and cleaved caspase‐3 with treatment of ATX (5, 10, 20 μmol/L) decreased distinctly (*P* < .01). The above data further indicated that ATX has a protective effect against OGD‐induced apoptosis (*P* < .05).

### 3.4 ATX modulates mitochondrial membrane potential (Δψm)

Fluorescence intensities of rhodamine 123 displayed the number of cells in different states of Δψm. Cells in the control group were mostly under high Δψm, whereas the percentage of high‐Δψm cells decreased to 39.6% by OGD. ATX (5, 10, and 20 μmol/L) significantly improved the Δψm in cells of dose groups in a dose‐dependent manner (*P* < .05, Figure 4C).

### 3.5 ATX is a potential Nrf2 activator in SH‐SY5Y cells

**FIGURE 5:**
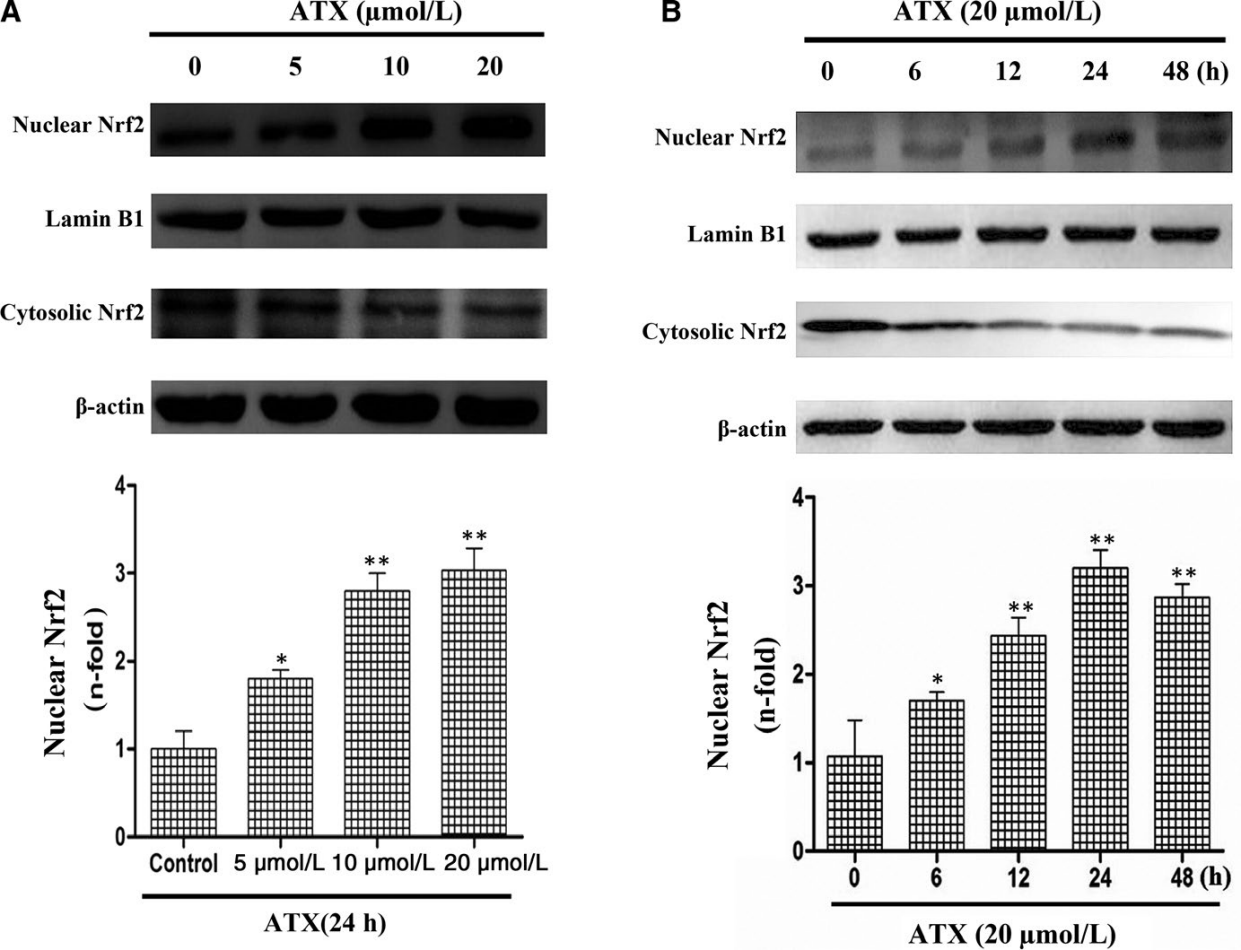
Effect of ATX on Nrf2 translocation in human SH‐SY5Y cells. A, SH‐SY5Y cells were treated with different concentrations of ATX (5, 10 and 20 μmol/L) for 24 h. B, SH‐SY5Y cells were treated with ATX (20 μmol/L) with different time durations (6, 12, 24 and 48 h). The nuclear and cytosolic levels of Nrf2 were determined by Western blot analysis. Lamin B1 and β‐actin were used as nuclear and cytosolic loading controls, respectively. Results were expressed as mean ± SD (n = 3). *P < .05, **P < .01 vs Control group. Significance was determined by one‐way analysis of ANOVA followed by Dunnett's test

Cells were treated either with ATX (5, 10, 20 μmol/L) for 24 hours or with ATX (20 μmol/L) for 6, 12, 24 and 48 hours. As shown in Figure 5A, Nrf2 evidently translocated into the nucleus in a dose‐dependent manner. In addition, the Nrf2 protein decreased in cytoplasm (*P* < .05). Compared with the control group, Nrf2 protein level in nucleus increased in a time‐dependent manner (*P* < .05, Figure 5B). The abundance of Nrf2 protein in cytoplasm decreased in a time‐dependent manner (*P* < .05). These results highlighted that ATX may be a potential Nrf2 activator by demonstrating its ability to stimulate Nrf2 transcription.

### 3.6 ATX modulates the activation of the PI3K/Akt/GSK3β/Nrf2 pathway and protective effects against OGD‐induced cytotoxicity

**FIGURE 6:**
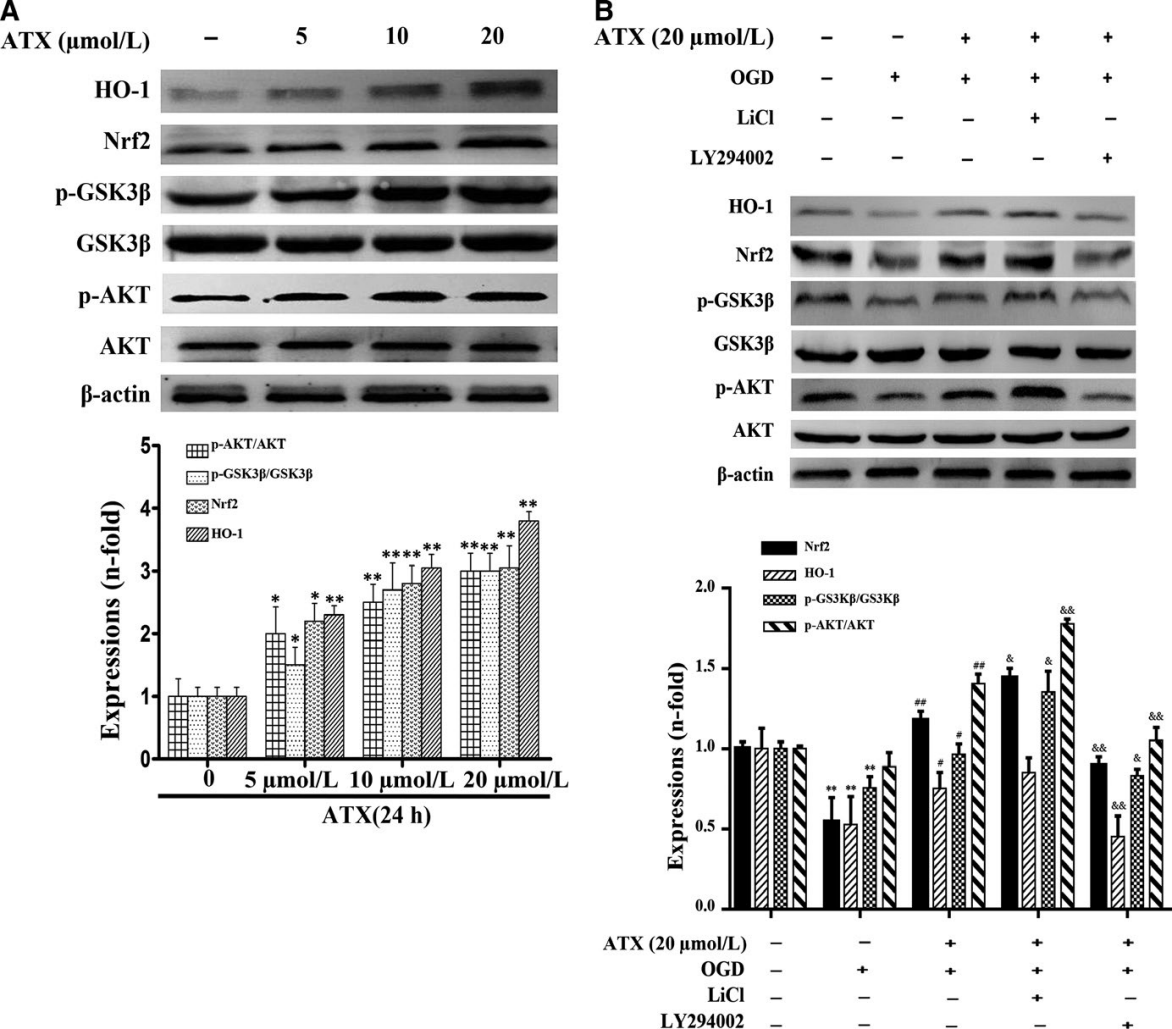
Effect of ATX on protein expressions of AKT, p‐Akt, GSK3β, p‐GSK3β, nuclear Nrf2 and HO‐1. (A) SH‐SY5Y cells were treated with ATX (5, 10 and 20 μmol/L) for 24 h, and cell lysates were subjected to immunoblot analysis for detecting the levels of AKT, p‐Akt, GSK3β, p‐GSK3β, nuclear Nrf2 and HO‐1. β‐actin was used as cell lysates loading controls. B, SH‐SY5Y cells were pretreated with a selective GSK3β inhibitor (LiCl, 10 μmol/L) and a selective PI3K/Akt inhibitor (LY294002, 10 μmol/L) for 1 h prior to incubation with ATX (20 μmol/L) for 24 h and OGD for 3 h. The densitometry analysis was shown below them. Results were expressed as mean ± SD (n = 3). *P < .05, **P < .01, vs Control group. #*P* < .05, ##*P* < .01, vs OGD group. &*P* < .05, &&*P* < .01 vs. ATX + OGD group. Significance was determined by one‐way analysis of ANOVA followed by Dunnett's test

To explore whether ATX activates Nrf2 signalling via the PI3K/Akt/GSK3β‐dependent pathway, the variations of protein expression in SH‐SY5Y cells were measured by Western blot. Our results in Figure 6A showed that ATX (5, 10, 20 μmol/L) significantly increased HO‐1 and Nrf2 expressions, as well as p‐Akt/Akt and p‐GSK3β/GSK3β ratios compared with the control group (*P* < .05).

Compared with control group, OGD inhibited HO‐1 expressions when p‐Akt/Akt and p‐GSK3β/GSK3β ratios were simultaneously decreased (*P* < .01, Figure 6B). However, ATX showed its protective properties by markedly inhibiting HO‐1, Nrf2, p‐Akt/Akt and p‐GSK3β/GSK3β decreasing effects caused by OGD (*P* < .05, Figure 6B).

To further support our hypothesis that the mechanism of ATX‐mediated protection is via the activation of the PI3K/Akt/GSK3β/Nrf2 signalling pathway, the GSK3β inhibitor LiCl and PI3K/Akt inhibitor LY294002 were employed in the study. Compared with ATX (20 μmol/L) + OGD treatment group, LY294002 (10 μmol/L, 1 hours) significantly decreased p‐Akt/Akt and p‐GSK3β/GSK3β ratios, and inhibited Nrf2 nuclear localization and HO‐1 protein expression (*P* < .01, Figure 6B); HO‐1, p‐Akt/Akt and p‐GSK3β/GSK3β increased on LiCl (10 μmol/L, 1 hour) exposure (*P* < .05, Figure 6B). Nrf2 translocation was promoted by LiCl treatment (*P* < .05, Figure 6B). These data suggested that ATX protects SH‐SY5Y cells against OGD‐induced cytotoxicity via the PI3K/Akt/GSK3β/Nrf2 signalling pathway.

## 4 DISCUSSION

These results suggest that ATX conspicuous inhibited the OGD damage of SH‐SY5Y cells in a dose and time‐dependent manner. The mechanisms of this phenomenon may be related to involve upstream control of Nrf2 through PI3K/Akt/GSK3β pathway in SH‐SY5Y cells. Above research, results provide a mechanistic basis for the potential application of ATX as a candidate therapeutic for ischaemic stroke, since previous studies have reported that the SH‐SY5Y cells under OGD damage have been employed as a simulation to ischaemia.^7^, ^13^

More and more experimental evidences show that oxidative stress, defined as an excess production of ROS, plays critical roles in the pathogenesis of cerebral ischaemia.^14^, ^15^ Excess ROS levels can disrupt the structures of lipids, proteins, and DNA and induce cell death.^16^, ^17^ When cells are suffering oxidative damage, ROS could undergo lipid peroxidation reaction with biological macromolecules such as polyunsaturated fatty acids and nucleic acids related to phospholipid, enzymes and membrane receptors of bio‐membrane, producing lipid peroxidation products, which could affect structures and functions of organelles.^18^, ^19^ In this study, our data show that ATX can significantly reduce the accumulation of intracellular ROS in a dose‐dependent manner. MDA is a by‐product of the oxidation of polyunsaturated fatty acids; therefore, it serves as a marker of oxidative stress. SOD is a member of the superoxide dismutase family, which converts superoxide radicals in the mitochondria to less toxic agents.^20^ In our study, MDA levels with OGD significantly increased whereas SOD levels decreased. Hence, treatment with ATX up‐regulated SOD expression and decreased ROS and MDA levels, thereby displaying antioxidant properties.

Apoptosis is known to participate in numerous biological processes, such as maintenance of tissue homeostasis. Dysfunction of apoptosis has been implicated in various human diseases, including neurodegenerative diseases and cerebral ischaemia.^21^ The ratio of Bcl‐2/bax was considered as an index of cell apoptotic states, since Bcl‐2 was proved to prevent oxidative damage to cellular constituents whereas its conserved homolog bax could heterodimerize with it and disable the protective effects of Bcl‐2.^22^ Cleaved caspase‐3, known as caspase‐3 p17, was one of the four subunit components departed from the original form of caspase‐3 (p32) protein when caspase‐3 was activated.^23^ Activated caspase‐3 could cleave DNA‐dependent protein kinases, thereby interfere replication and transcription of the DNA which results in cell apoptosis.^24^ Our previous research demonstrated that ATX has anti‐apoptotic properties in cerebral ischaemia.^3^ In this study, ATX prevented the OGD‐induced high expression of apoptotic factors Bax and cleaved caspase‐3 but restored Bcl‐2 and caspase‐3 expression. Mitochondria, as one of the main targets for screening anti apoplexy drugs, also play a key role in apoptotic cell death.^25^, ^26^ The loss of Δψm is treated as a pivotal apoptotic characteristic. In our study, ATX prevents OGD‐induced apoptosis of SH‐SY5Y cells by changing mitochondrial membrane permeability. Besides, our findings have shown that ATX can protect SH‐SY5Y cells from OGD‐induced oxidative damage and apoptotic death.

**FIGURE 7:**
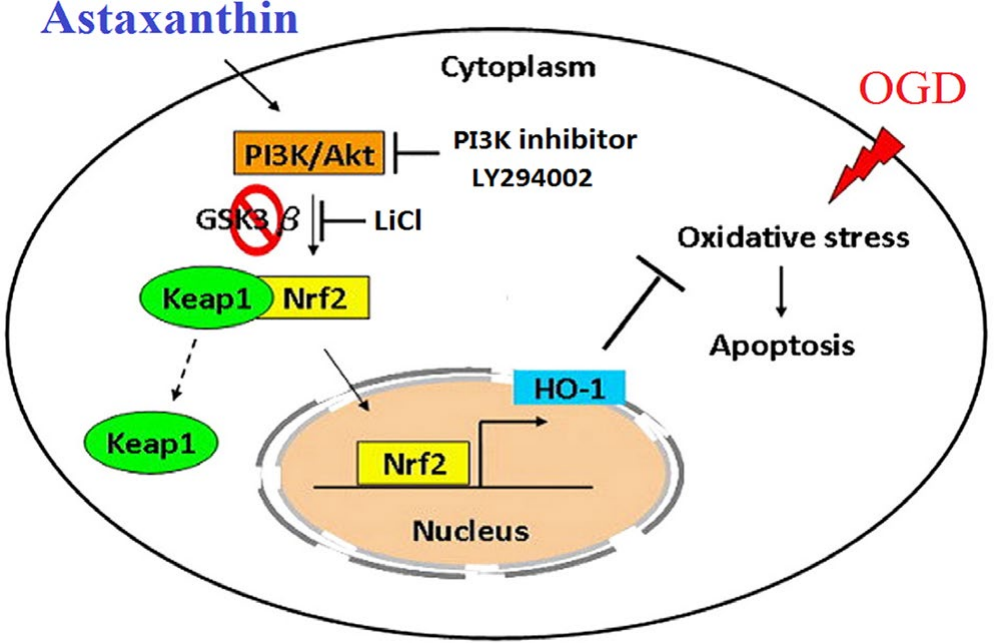
Proposed mechanisms for the protective effects of ATX against OGD‐induced apoptosis. The antiapoptotic effect of ATX is through the inhibition of oxidative stress. Our results demonstrated that up‐regulation of PI3K/Akt signaling‐mediated GSK3β inhibition enhances Nrf2 expression and nuclear translocation, which further activate downstream antioxidant enzyme expression. This activation is involved in the antioxidant action of ATX on OGD‐triggered oxidative damage in SH‐SY5Y cells

Nrf2 plays a key role in protecting cells from various kinds of invasion.^27^ Furthermore, Nrf2 is also a basic leucine zipper and redox‐sensitive transcription factor, which plays an important role in the regulation of antioxidant genes. In response to oxidative stress, Nrf2 will be dissociated from Keap1 and lead to nuclear translocation, which provides signals for the transcriptional regulation of ARE‐related genes, including HO‐1.^28^, ^29^ As the result of this study shows, ATX can increase the nuclear translocation of Nrf2. The potential molecular mechanism mediated by ATX has been the focus of extensive research. Among multifarious upstream signalling pathways, Nrf2 is the target of PI3K/Akt and GSK3β protein. P‐Akt and p‐GSK3β can promote the separation of Nrf2 from Keap1, leading to the translocation of Nrf2 into the nucleus. Here we proposed that PI3K/Akt/GSK3β signalling pathway may be the regulator of cell survival after ATX treatment. The PI3K/Akt signalling pathway is a typical anti‐apoptotic signalling pathway, and its activity can be regulated by redox.^30^ LiCl is a common GSK3β dephosphorization inhibitor, which could activate PI3K and increase the phosphorylation of proteins downstream, such as the Akt and GSK3β, as well as Nrf2 translocation, and prevents hypoxia cells from apoptosis.^11^, ^31^ In addition, p‐GSK3β participates in the protective cellular response to oxidative stress.^32^ Moreover, LY294002 could down‐regulate Akt activation and Nrf2 translocation by especially inhibiting the activity of PI3K upstream.^33^ In this study, we found that the activated p‐Akt and p‐GSK3β levels in ATX treated cells were higher than those in untreated cells. To further support this pathway's mechanistic value to ATX’s properties, LY294002 inhibited the increasing effect of p‐Akt protein expressions by ATX and LiCl facilitated Nrf2 nuclear localization and increased HO‐1 protein expression. It should be emphasized that ATX played a similar role to LiCl in inhibiting the dephosphorylation of GSK3β and promoting the translocation of Nrf2 into the nucleus. These inhibitors proved ATX's protective effect against OGD‐induced cell apoptosis (Figure 7). Thus, we have shown that the PI3K/Akt/GSK3β antioxidant defence pathway plays a critical role in ATX's cytoprotective effect.

Although in our research, we found that ATX had the ability to decrease OGD damage in SH‐SY5Y cells by activating PI3K/Akt/GSK3β/Nrf2 signalling pathway, it must be emphasized that in addition to Nrf2, whether other redox‐sensitive transcription factors such as AP‐1 and NF‐κB can regulate the expression of antioxidant enzymes.^34^ Therefore, ATX may also induce Nrf2 transport from cytoplasm to nucleus through other signalling pathways, where Nrf2 is regarded as an important regulator, such as p38 MAPK, AMPK, ERK1/2,^35^, ^36^ whereas the mechanisms still require further investigation.

## 5 STATISTICAL ANALYSIS

All blotting images were acquired and processed by ChemiCapture software, quantified by ImageJ and image‐gathered by Photoshop. All values of this study are presented by mean ± SD. Variations of parameters between control and dose groups were analysed by one‐way analysis of ANOVA followed by Dunnett's test. A level of *P* < .05 was considered statistically significant.

## CONFLICT OF INTEREST

The authors declare no competing financial interests.

## AUTHOR CONTRIBUTIONS

**Jie Zhang:** Conceptualization (equal); data curation (equal); formal analysis (equal); funding acquisition (equal); investigation (equal); methodology (equal); project administration (equal); writing‐original draft (equal). **Changling Ding:** Conceptualization (equal); data curation (equal); formal analysis (equal); funding acquisition (equal); investigation (equal); methodology (equal); project administration (equal). **Shuping Zhang:** Data curation (equal); funding acquisition (equal); project administration (equal); resources (equal); software (equal); supervision (equal); validation (equal); visualization (equal); writing‐review and editing (equal). **Yangyang Xu:** Data curation (equal); formal analysis (equal); funding acquisition (equal); methodology (equal); project administration (equal); resources (equal); software (equal); supervision (equal); validation (equal); visualization (equal); writing‐original draft (equal); writing‐review and editing (equal).

## Data Availability

The data that support the findings of this study are available from the corresponding author upon reasonable request.
